# Anti-tumor necrosis factor _V_NAR single domains reduce lethality and regulate underlying inflammatory response in a murine model of endotoxic shock

**DOI:** 10.1186/1471-2172-14-17

**Published:** 2013-04-02

**Authors:** Rafael Bojalil, María Teresa Mata-González, Fausto Sánchez-Muñoz, Yepci Yee, Iván Argueta, Lucía Bolaños, Luis Manuel Amezcua-Guerra, Tanya Amanda Camacho-Villegas, Edna Sánchez-Castrejón, Walter Jakob García-Ubbelohde, Alexei Fedorovish Licea-Navarro, Ricardo Márquez-Velasco, Jorge Fernando Paniagua-Solís

**Affiliations:** 1 Department of Immunology, Instituto Nacional de Cardiología Ignacio Chávez, Mexico City, Mexico; 2 Department of Health Care, Universidad Autónoma Metropolitana-Xochimilco, Mexico City, Mexico; 3 Laboratorios Silanes, S. A. de C. V. Amores 1304, Col del Valle, Mexico City, 03100, Mexico; 4 Marine Science Faculty, Universidad Autónoma de Baja California, Km. 103 Carretera Tijuana-Ensenada, Ensenada, Mexico; 5 Marine Biotechnology Department, Centro de Investigación Científica y de Educación Superior de Ensenada (CICESE), Ensenada, Mexico; 6 Department of Health Sciences, Universidad del Valle de México, Mexico City, Mexico; 7 Current Address (JFP-S): Teraclon IDF; Parque Científico de Madrid, Tres Cantos, Madrid, España

**Keywords:** Endotoxic shock, Sepsis, Anti-TNF, _V_NAR, Inflammation

## Abstract

**Background:**

In sepsis, tumor necrosis factor (TNF) is the key factor triggering respiratory burst, tissue injury and disseminated coagulation. Anti-TNF strategies based on monoclonal antibodies or F(ab’)_2_ fragments have been used in sepsis with contradictory results. Immunoglobulin new antigen receptors (IgNAR) are a unique subset of antibodies consisting of five constant (_C_NAR) and one variable domains (_V_NAR). _V_NAR domains are the smallest, naturally occurring, antibody-based immune recognition units, having potential use as therapy.

Our aim was to explore the impact of an anti-TNF _V_NAR on survival in an experimental model of endotoxic shock. Also, mRNA expression and serum protein of several inflammatory molecules were measured.

**Results:**

Endotoxic shock was induced by lipopolysaccharide (LPS) in male Balb/c mice. Animals were treated with anti-TNF _V_NAR domains, F(ab’)_2_ antibody fragments, or saline solution 15 minutes before, 2 h and 24 h after lethal dose_100_ (LD_100_) LPS administration. TNF blockade with either _V_NAR domains or F(ab’)_2_ fragments were associated with lower mortality (60% and 75%, respectively) compared to LD_100_. Challenge with LPS induced significant production of serum TNF and interleukins -10 and -6 at 3 h. After that, significant reduction of IL-6 at 24 h (vs 3 h) was shown only in the _V_NAR group. Nitrites level also increased in response to LPS.

In liver, TNF and IL-10 mRNA expression showed a pro-inflammatory imbalance in response to LPS. Blocking TNF was associated with a shift towards an anti-inflammatory status; however, polarization was more pronounced in animals receiving F(ab’)_2_ fragments than in those with _V_NAR therapy. With regard to IL-6, gene expression was increased at 3 h in all groups. TNF blockade was associated with rapid and sustained suppression of IL-6 expression, even more evident in the _V_NAR group. Finally, expression of inducible-nitric oxide synthase (iNOS) increased in response to LPS at 3 h, but this was decreased at 24 h only in the anti-TNF _V_NAR group.

**Conclusions:**

Anti-TNF _V_NAR single domains improved survival in a murine model of endotoxic shock. Protection was associated with regulation in the TNF/IL-10 balance, attenuation of IL-6 and iNOS gene expression in the liver as well as decreased serum IL-6 concentration.

## Background

Sepsis is a life-threatening condition which results from an exacerbated inflammatory process caused by complex interactions between the innate immune system and either bacterial sources of infection or their circulating antigens, mainly lipopolysaccharides (LPS). Circulating LPS is engaged by Toll-like receptor 4 (TLR4) on the surface of monocytes and macrophages, which in turn result in cell activation and early production of large amounts of inflammatory cytokines [1,2]. Tumor necrosis factor (TNF) plays a key role in sepsis due to its ability to trigger respiratory burst and nitric oxide production while diminishing peripheral vascular resistance, leading to disseminated intravascular coagulation and multiple organ failure [3-5].

Elucidation of pathophysiological events that underlie sepsis has led to the development of molecules targeting these pathways. Major strategies to block TNF have included the use of anti-TNF monoclonal antibodies, soluble TNF receptors with IgG chimeric protein, and anti-TNF F(ab’)_2_ fragments [6-8]. Interestingly, both the effectiveness and usefulness of each anti-TNF strategy varies among studies, often being conflictive and even contradictory. This could be partly the result of the intensity of TNF blockade as well as the tissue penetration achieved by each type of molecule.

Immunoglobulin new antigen receptors (IgNAR) are a unique subset of antibodies found in sharks. It consists of homodimers of polypeptide chains, each comprising a single variable (_V_NAR) and five constant (_C_NAR) domains. Single _V_NAR domains are candidates to be the smallest, naturally occurring, antibody-based immune recognition units [9]. Moreover, _V_NAR domains have been suggested as a highly effective set of molecules capable to access antigenic sites rarely targeted by conventional antibody-based strategies, positioning them as attractive candidates for therapy [10-12]. Thus, we explored the survival of mice in an experimental model of endotoxic shock, after treatment with a _V_NAR single domain directed against TNF.

## Results

### Efficacy of _V_NAR on survival

**Figure 1:**
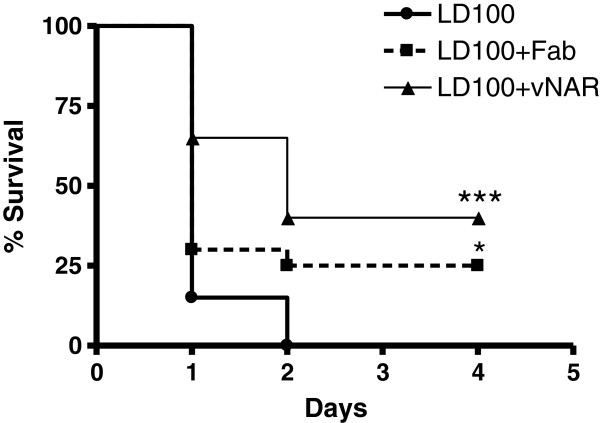
**Survival of animals treated with LD**_**100 **_**of LPS. **The figure is a compilation of two independent assays with animals treated with _V_NAR single domains Anti-TNF or Fragments F(ab)^2^ anti-TNF. Each group included 20 animals. Survival comparisons were analyzed with the Mantel-Haenzel Log Rank test, differences were considered significant when * p < 0.05, ***p < 0.001 vs control.

Survival of mice was documented for up to four days after induction of endotoxemia (Figure 1). All animals in the endotoxemic shock group died within 48 h following administration of LD_100_ LPS. In contrast, TNF blockade with either _V_NAR domains or F(ab’)_2_ fragments reduced mortality as early as 24 h and continued until 96 h surveillance. _V_NAR domains were associated with lower mortality compared with F(ab’)_2_ fragments, although this was not significant (40% versus 25% survival; p = 0.16), however both groups were significantly protected vs LD_100_ treatment (p < 0.001 and 0.05 respectively).

### Inflammatory markers in sera

**Figure 2:**
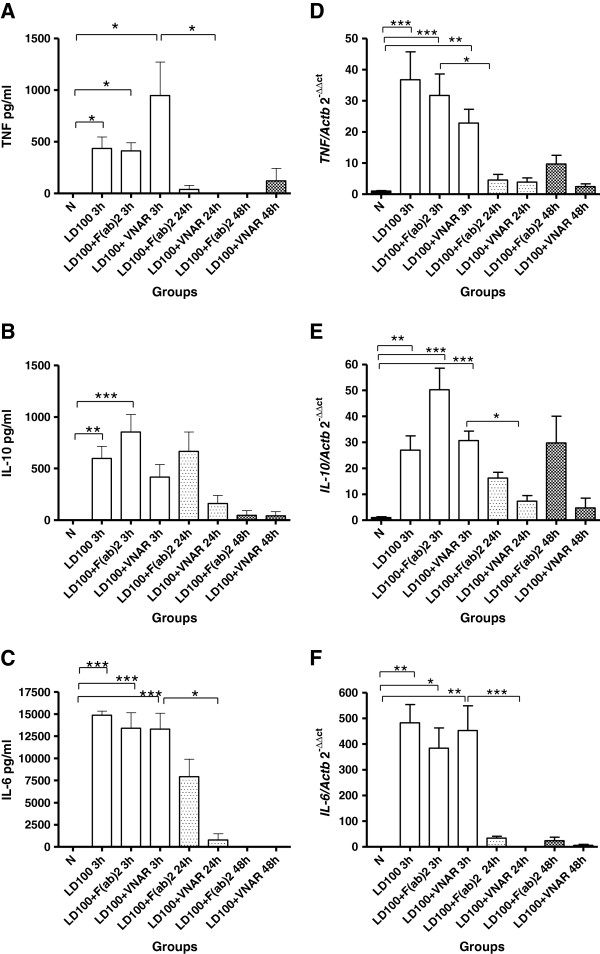
**Production and expression of TNF, IL-6 and IL-10. **Panels **A**, **B**, and **C **show levels of cytokines in serum of groups LD_100_, LD_100_ + _V_NAR or LD_100_ + F(ab)^2 ^at different times; while panels **D**, **E**, and **F **show the expression in liver of the same cytokines at same groups and times; data represent mean ± standard error. The differences among groups were determined using a Kruskal-Wallis test and *post hoc *analysis using a Dunn’s multiple comparison test; differences were considered significant when *p < 0.05, **p < 0.01, ***p < 0.001. N = 10 animals for each group and time point.

Induction of endotoxic shock produced a significant increase in the serum concentration (mean ± standard error) of TNF at 3 h after LPS administration (Figure 2, panel A) in the untreated group (435 ± 112 pg/mL), F(ab’)_2_ group (411 ± 79 pg/mL), and _V_NAR group (947 ± 324 pg/mL) compared to animals with neither administration of LPS nor anti-TNF (0 pg/mL; p < 0.05 for all comparisons). Nevertheless, F(ab’)_2_ fragments induced a persistent decrease of TNF at 24 h (38 ± 38 pg/mL) and 48h (0 pg/mL), while _V_NAR domains produced a transitory decrease at 24 h (0 pg/mL; p < 0.05 vs _V_NAR 3 h), with a subsequent mild increase at 48 h (121 ± 121 pg/mL). Endotoxemic shock was also associated with an early raise of IL-10 (Figure 2, panel B) in untreated (598 ± 116 pg/mL; p < 0.01 vs. animals with no endotoxemia), treated with anti-TNF F(ab’)_2_ (854 ± 169 pg/mL; p < 0.001) and with _V_NAR groups (418 ± 119 pg/mL). After 24 h both anti-TNF treatments induced a similar decline in the IL-10 concentration.

TNF/IL-10 ratio was considered to represent antagonistic inflammatory responses in endotoxic shock [13]. While a predominance of anti-inflammatory response driven by IL-10 was steadily observed in the F(ab’)_2_ group from 3 h to 48 h of surveillance, _V_NAR treated animals showed a pro-inflammatory response characterized by high TNF and low IL-10 serum concentrations.

Serum concentrations (mean ± standard error) of IL-6 were similar at 3 h in all endotoxemic shock groups (Figure 2, panel C) and significantly different to those of normal group (p < 0.001). F(ab’)_2_ group showed a gradual decrement (from 13392 ± 1749 to 7919 ± 1991 pg/mL at 24 h), while this decline was abrupt in the _V_NAR group (from 13288 ± 1793 to 783 ± 703 pg/mL; p < 0.05). IL-6 levels were undetectable at 48 h in all groups.

**Figure 3:**
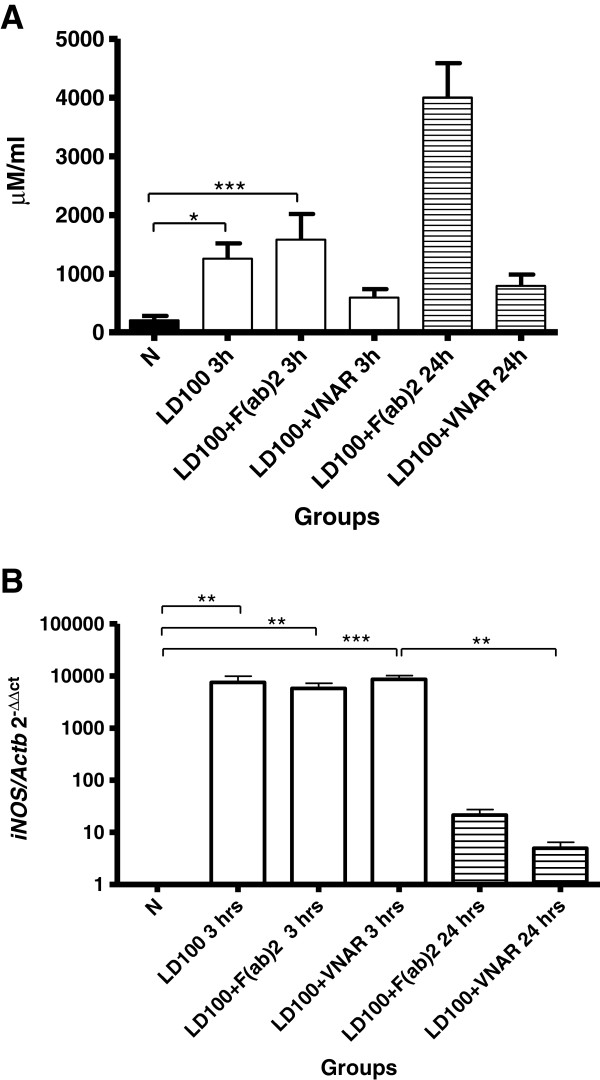
**Production of NO**_**2**_^**- **^**and expression of iNOS. **Panel **A **shows levels of NO_2_^-^ in serum of groups LD_100_, LD_100_ + _V_NAR or LD_100_ + F(ab)^2 ^at different times; panel **B **shows expression of iNOS in the same groups and times; data represent mean ± standard error. Differences among groups were determined using a Kruskal-Wallis test and *post hoc *using a Dunn’s multiple comparison test; differences were considered significant when *p < 0.05, **p < 0.01, **p < 0.001. N = 10 animals for each group and time point.

Nitrites concentration (mean ± standard error) in sera was found to be elevated at 3h in LD_100_ (1256 ± 262 μM/mL)(p < 0.05) and F(ab)2 (1581 ± 438 μM/mL) (p < 0.001) groups vs normal group (206 ± 74 μM/mL), but not in _V_NAR group (592 ± 144 μM/mL) (Figure 3, panel A). While their concentration remained almost unchanged at 24h (793 ± 189 μM/mL) in the anti-TNF _V_NAR group, it increased around three-fold in the F(ab’)_2_ fragments group (from 1581 ± 438 to 4000 ± 657 μM/mL).

### Liver mRNA quantification

Relative mRNA expression of TNF and IL-10 in liver increased at 3 h as a result of LPS administration (Figure 2, panels D and E, **p < 0.01, ***p < 0.001). However while LD_100_ of LPS induced a predominant pro-inflammatory response, TNF blockade with either molecule was associated with an anti-inflammatory predominance. However, subtle differences in the TNF/IL-10 balance were found, because polarization to an anti-inflammatory status was more pronounced in the animals receiving F(ab’)_2_ fragments than in those with _V_NAR therapy.

Interleukin-6 gene expression in liver increased early (3 h) after LPS challenge in all groups (Figure 2, panel F). TNF blockade was associated with rapid (24 h) and sustained (48 h) suppression of IL-6 mRNA expression; however, the intensity was different according to the anti-TNF strategy used. Indeed, _V_NAR therapy showed to be more effective than F(ab’)_2_ to suppress IL-6 mRNA expression at 24 h (1.33 versus 34.27; p < 0.05) and 48 h (5.69 versus 24.20 p < 0.05) after induction of endotoxemia.

Finally, expression of mRNA inducible-nitric oxide synthase (iNOS) in liver was similar between all groups at 3 h (Figure 3, panel B) (from 5811 ± 1422 to 8560 ± 1603); at 24 h both anti-TNF schemes strikingly decreased iNOS mRNA expression, but only in _V_NAR group this reduction was found to be statistically significant.

## Discussion

_V_NAR domains are promissory antibody-based molecules because of their particular features such as low molecular weight and size, resistance to gastric pH and long CDR3 loop. _V_NAR domains are currently used to neutralize bioactive molecules and viruses [11,14], as well as to perform *in vitro* diagnostic assays [12]. Herein, we present the first study aimed to report an anti-TNF _V_NAR that improves survival in a murine model of endotoxic shock. Our results support a role for TNF bioactivity blockade in the treatment of sepsis, and suggest that different anti-TNF strategies may reach different survival rates through differentially attenuating inflammatory mechanisms.

In the present study, anti-TNF administration was not associated with early (3 h) depletion of serum TNF levels; on the contrary, TNF was detected in sera from animals in all groups. In contrast, serum TNF declined to almost undetectable levels at 24 h and 48 h after administration of either F(ab’)_2_ fragments or _V_NAR domains. These results are opposed to others studies showing that treatments with anti-TNF antibodies early reduce serum TNF concentration [15,16]. A possible explanation is based on the molecular weight and bioavailability of each molecule. In this context, while conventional murine monoclonal IgG antibodies (159 kD) have a serum half-life of 25 days [17], mean serum half-life of smaller molecules is reduced, possibly as a result of improved clearance. Indeed, F(ab’)_2_ fragments (100 kD) show a mean serum half-life of 2 hrs [18], and _V_NAR (14 KD) single domains as little as 3 min to 1.5 hrs [19].

The liver plays key physiological roles including blood filtering of toxins as well as inactivation and clearing of bacterial antigens and products [20]. Thus, the liver is a site in which early inflammatory changes in sepsis can be assessed by measuring the expression of several pro- and anti-inflammatory molecules [21]. Due to the size of _V_NAR single domains, they may arrive to the liver and, possibly, deeply penetrate in hepatic tissue [22], which ultimately may result in the attenuation of inflammatory response. In our study, untreated animals with endotoxic shock showed an intense pro-inflammatory response featured by high expression of TNF and low expression of IL-10 in liver, while anti-TNF treated groups were characterized by an anti-inflammatory response featured by high expression of IL-10 and low expression of TNF.

The protective role of IL-10 in animal sepsis may be due to its antagonistic effect on the production and overall functioning of TNF [23-25]. In this way, it has been demonstrated that neutralization of IL-10 in septic animals and the induction of endotoxemic shock in IL-10 *knockout* animals are associated with increased tissue injury [26,27] and higher serum levels of interferon-γ and TNF [28,29]. In our study, a lower anti-inflammatory TNF/IL-10 ratio was associated with the administration of _V_NAR domains compared to F(ab’)2 fragments. This could be associated with the trend for higher survival observed in the group on _V_NAR therapy. In support to this notion, we have previously described that regulation in the IL-1β/IL-10 balance is associated with protection against lethality in a sepsis model of cecal ligation and puncture [7,30]. The presence of similar results in different models of sepsis further supports that modulation of inflammation would tame tissue injury mechanisms, while full blockade of an inflammatory pathway would facilitate polarization of the immune response in either systemic inflammatory response syndrome or its counterpart, compensatory anti-inflammatory response syndrome [7].

Tumor necrosis factor directly influences the production of IL-6 and iNOS [31-33] and it is conceivable that its attenuation could have regulated both gene expression and serum concentration of IL-6 and nitric oxide (NO) in our experiments. For example, it has been reported that the administration of anti-IL-6 antibodies improves survival in sepsis [34,35]; while TNF blockade inhibits hepatic expression of iNOS and nitrotyrosine in mice with endotoxic shock [36].

In addition, protection of liver seems to be critical to obtain beneficial outcomes in sepsis. In a recent study performed in rats with polymicrobial sepsis, treatment with hyperoncotic albumin attenuates hepatic injury in association with reduced plasma levels of IL-1β, IL-6, liver enzymes, and O_2_^-^ concentrations [37]. These results support anti-TNF _V_NAR domains as an alternative approach in the treatment of sepsis, due to its attenuating effects on the inflammatory response showed in liver; however these must be further studied in more aggressive models such as the polymicrobial sepsis induced by cecal ligation and puncture. Related to the latter study, a recent rat gut model of indomethacin-induced jejunoileitis showed that anti-TNF-α monoclonal antibody reduced iNOS expression and IL-1 beta, the latter two thought to be key mediators of inflammatory bowel disease [38].

## Conclusions

Anti-TNF _V_NAR single domains are a novel strategy useful to improve survival in a murine model of endotoxic shock, with efficacy similar to that observed with the use of anti-TNF F(ab’)2 fragments. Protection against lethality was associated with regulation in the TNF/IL-10 inflammatory balance, attenuation of IL-6 and iNOS liver expression, and decreased IL-6 serum concentration.

## Methods

### Isolation of anti-TNF _V_NAR single domains

Isolation of anti-TNF _V_NAR domains was performed as previously described [39,40]. In brief, a specimen of horn shark *Heterodontus francisci* was repeatedly immunized (intravenous route) with human recombinant TNF (Peprotech Inc, Connecticut USA). After immunization, RNA from the spleen was obtained and used to amplify variable genes through polymerase chain reaction (PCR), and libraries were generated. A specific clone was obtained by phage display technique and cultures of *Pichia pastoris* were used to express it, in accordance with the Easy Select Pichia Expression Manual (Invitrogen, USA); pPICZαAvector and X-33 strain were used. vNAR single domains were isolated and purified in the Centro de Investigación Científica y de Educación Superior de Ensenada (Mexico).

### Induction of murine endotoxic shock and treatment schedule

Protocol was approved by the ethics committee at the Instituto Nacional de Cardiología Ignacio Chávez (Protocol number 11–726). Male Balb/c mice, classified as an inbred based on 20 or more successive brother-sister matings [41], 8–12 weeks old were housed under standard laboratory conditions with food and water *ad libitum* (UAM-Xochimilco, Mexico City, Mexico). Initially, independent experiments were performed to investigate the Lethal Dose 100 (LD_100_), which was found to be 20 mg/kg of LPS serotype O55:B5 (Sigma-Aldrich, St. Louis, MO) diluted in 100 μL 0.9% saline solution, administered by intraperitoneal (IP) injection in a single dose. Similarly, a pilot assay was performed to investigate the dose of anti-TNF _V_NAR domains useful to improve survival in the murine model of endotoxemic shock, doses of 0.01, 0.1 and 1 mg/kg of VNAR anti-TNF were tested, the latter dose was the only one that induced protection. To compare active treatment with anti-TNF _V_NAR domains, we used anti-TNF F(ab’)_2_ antibody fragments (Laboratorios Silanes, Mexico City, MX). We used the same dose of the latter antibody (1 mg/kg) since it had previously shown protective activity in the cecal ligation and puncture model.

Mice (20 per group) were allocated in groups to receive (A) LD_100_ LPS in 100 μL 0.9% saline solution by IP injection, these mice also received 100 μl of 0.9% saline solution by IP injection, 15 minutes before as well as at 2 and 24 h after LD_100_ LPS was administrated; (B) 1 mg/kg anti-TNF _V_NAR domains diluted in 100 μl 0.9% saline solution by IP injection, 15 minutes before as well as at 2 and 24h after LD_100_ LPS was administrated; (C) 1 mg/kg anti-TNF F(ab’)^2^ antibody fragments diluted in 100 μL 0.9% saline solution by IP injection administrated in a similar time schedule than _V_NAR domains; (D) 100 μl 0.9% saline solution IP in the same time schedule as the other groups; these mice received neither LPS nor anti-TNF treatment.

### Measurement of serum markers

Using another batch of mice, blood from 10 anesthetized animals for each group and time was obtained by cardiac puncture at 3, 24 and 48 h after induction of endotoxemia. Serum TNF, IL-6 and IL-10 concentrations were measured by enzyme-linked immunosorbent assays (R&D Systems, Minneapolis, MN). In addition, non-hemolysed serum was used to measure nitrites (NO_2_^-^) by means of a Griess reagent system (Promega Corp, Madison, USA). All assays were performed according to manufacturer.

### Liver mRNA quantification by RT-qPCR

Ten animals per group and time were sacrificed at 3, 24 or 48 h after LPS administration and liver tissue was immediately frozen and stored at -70°C. One-hundred milligrams from each liver were homogenized using Tri Reagent (Sigma-Aldrich). RNA integrity was assessed by agarose gel stained with ethidium bromide and purity was determined by spectrophotometer (260/280 > 1.8); 2 micrograms of total RNA were retro-transcribed using random primers in a 20 μl reaction with the Transcriptor First cDNA synthesis kit (Roche Applied Science, Indianapolis, USA). One microliter of cDNA was amplified by qPCR using the LightCycler 2.0 with LightCyclerTaqMan Master Mix (Roche) and PCR primers in combination with LNA hydrolysis probes designed with the probe finder software v. 2.45 from the Universal Probe Library Mouse Set (Roche).

PCR was performed using the following primers: Inducible nitric oxide synthase 2 (NM_010927.3), forward 5′-gggctgtcacggagatca-′3, reverse 5′-ccatgatggtcacattctgc-′3; IL-6 (NM_031168.1), forward 5′-gctaccaaactggatataatcagga-′3, reverse 5′-ccaggtagctatggtactccagaa-′3; IL-10 (NM_007393.3), forward 5′-cagagccacatgctcctaga-′3, reverse 5′-gtccagctggtcctttgttt-′3; TNF (NM_013693.2), forward 5′-tcttctcattcctgcttgtgg-′3, reverse 5′-ggtctgggccatagaactga-′3; and Actb (NM_007393.3), forward 5′-ctaaggccaaccgtgaaaag-′3, reverse 5′-accagaggcatacagggaca-′3 as a constitutive gen.

Relative expression of each gene was calculated according to 2^-ΔΔCt^. Assays were tested for linearity and reproducibility (variation coefficients <10%).

### Statistical analyses

Results are expressed as proportions or mean ± standard deviation. Comparison of survival curves was performed using the Mantel-Haenzel log-rank test. Differences between two independent groups were calculated by Mann–Whitney tests, while those comparisons including more than two groups were performed by the Kruskal-Wallis test (Dunn’s post test). All analyses were 2-tailed and a p < 0.05 value was used for significance. The GraphPad Prism v 4.02 statistical software (GraphPad Inc, San Diego, CA) was used.

## Abbreviations

IgNAR: Immunoglobulin new antigen receptors; IL: Interleukin; iNOS: Inducible nitric oxide synthase; LD100: Lethal dose 100; LPS: Lipopolysaccharide; TLR4: Toll-like receptor 4; TNF: Tumor necrosis factor; VNAR: Single variable domain.

## Competing interests

FS-M, YY, IA, LB and LMA-G declare they have no competing interest. RM-V and RB received a research support by Laboratorios Silanes-Fundación Mexicana para la Salud. MTM-G, WJG-U and JFP-S were employed by Laboratorios Silanes at the time of the manuscript was written. TAC-V, ES-C and AFL-N received a grant support by Laboratorios Silanes. Funding source was provided by Laboratorios Silanes and Fundación Mexicana para la Salud with the support fund number 471.

## Authors’ contributions

RM-V and RB contributed to the conception and design of the study, the interpretation of data and the elaboration of the manuscript. RM-V, BL, YY, AI, FS-M and LMA-G contributed with the development of experiments and in the interpretation of data. TAC-V, ES-C, WJG-U, AFL-N contributed in the isolation and production of anti-TNF _V_NAR. MTM-G, WJG-U, JFP-S contributed in the isolation, characterization, and production of anti-TNF _V_NAR. All authors reviewed and approved the final manuscript.
